# Nonlinear multi-magnon scattering in artificial spin ice

**DOI:** 10.1038/s41467-023-38992-7

**Published:** 2023-06-09

**Authors:** Sergi Lendinez, Mojtaba T. Kaffash, Olle G. Heinonen, Sebastian Gliga, Ezio Iacocca, M. Benjamin Jungfleisch

**Affiliations:** 1 Department of Physics and Astronomy, University of Delaware, Newark, DE 19716 USA; 2 Center for Advanced Microstructures and Devices, Louisiana State University, Baton Rouge, LA 70806 USA; 3 Materials Science Division, Argonne National Laboratory, Lemont, IL 60439 USA; 4 Swiss Light Source, Paul Scherrer Institute, 5232 Villigen PSI, Switzerland; 5 Department of Mathematics, Physics, and Electrical Engineering, Northumbria University, Newcastle upon Tyne, NE1 8ST United Kingdom; 6 Center for Magnetism and Magnetic Nanostructures, University of Colorado Colorado Springs, Colorado Springs, CO 80918 USA; 7 Present Address: Seagate Technology, 7801 Computer Ave., Bloomington, MN 55435 USA

**Keywords:** Nanoscience and technology, Magnetic properties and materials, Metamaterials, Ferromagnetism

## Abstract

Magnons, the quantum-mechanical fundamental excitations of magnetic solids, are bosons whose number does not need to be conserved in scattering processes. Microwave-induced parametric magnon processes, often called Suhl instabilities, have been believed to occur in magnetic thin films only, where quasi-continuous magnon bands exist. Here, we reveal the existence of such nonlinear magnon-magnon scattering processes and their coherence in ensembles of magnetic nanostructures known as artificial spin ice. We find that these systems exhibit effective scattering processes akin to those observed in continuous magnetic thin films. We utilize a combined microwave and microfocused Brillouin light scattering measurement approach to investigate the evolution of their modes. Scattering events occur between resonance frequencies that are determined by each nanomagnet’s mode volume and profile. Comparison with numerical simulations reveals that frequency doubling is enabled by exciting a subset of nanomagnets that, in turn, act as nanosized antennas, an effect that is akin to scattering in continuous films. Moreover, our results suggest that tunable directional scattering is possible in these structures.

## Abstract (web summary)

Artificial spin ices are composed of tiny magnets arranged in a lattice. Despite their simplicity, they exhibit rich dynamic magnetic behaviour. Here, Lendinez et al demonstrate that, like continuous magnetic thin films, artificial spin ices can exhibit non-linear magnon-magnon scattering which, in conjunction with their reconfigurability, offers great potential for tuneable magnon transport.

## Introduction

The field of magnonics^1^ aims to manipulate the fundamental magnetic excitations, magnons, for practical applications. Research into functional devices includes structures ranging from waveguides to periodic magnonic crystals^2^. In the former, magnon propagation and interference lead to functional outcomes, e.g., interferometers^3^, transistors^4^, and designer networks^5,6^. In the latter approach, periodic one-dimensional patterns are created to establish a superlattice used to engineer the desired magnon band structure^7,8^, similar to the approach taken in photonics and inspired by semiconductors^9^.

In two dimensions, artificial spin ices (ASIs), which are geometric arrangements of nanomagnets, can function as magnonic crystals^10–12^. The additional dimensionality comes with the added complexity of frustration: the existence of multiple local minima in the energy landscape^13^. Perhaps counterintuitively, such complexity also makes ASIs attractive as reconfigurable magnonic crystal^14^ whereby the magnons’ properties can be manipulated by way of the large range of magnetization states supported by the ASI system. Another important benefit of ASI is the vast number of possible geometries. These geometries, constrained only by nanofabrication limitations^15^, are already extending into three-dimensional structures^16–22^.

Despite significant progress in investigating dynamics in ASIs both theoretically^23–28^ and experimentally^24,29–40^, the detected modes have been limited to the system’s eigen-frequencies. Experimentally, this is achieved by microwave absorption via an antenna or by Brillouin light scattering (BLS) of the thermal modes^41,42^. This type of data leaves open critical questions on the fundamental features of magnons in ASIs: what is the coherence of magnons? How do magnons scatter? These questions are intimately related to the manifestation of quantum-mechanical effects at the mesoscale and their manipulation. Past works examined nonlinear magnon processes including nonlinear magnon scattering in structured ferromagnets in individual micro- and nanomagnets as well as arrays of nanomagnets^43–51^. However, nonlinearities in ASI structures consisting of a coupled array of two nonequivalent nanomagnet sublattices with different properties have not been examined so far.

Here, we present a combined microwave absorption and microfocused BLS measurement system to investigate the evolution of the modes in a square ASI lattice excited at a particular frequency and subject to an external magnetic field. We find that magnons in the ASI exhibit effective scattering processes akin to those observed in continuous magnetic films. Higher-order harmonics are detected by exciting the system with a strong microwave signal, and parametric pumping is achieved. These scattering events are determined by each nanomagnet’s mode volume and profile as well as the coupling between the dynamics of the two sublattices that exhibit dissimilar frequency-field behaviors. However, these modes can occupy different volumes within the nanomagnets so that their frequencies can be controlled by external excitation. Comparison with numerical simulations reveal an excitation mechanism unique to ASI, whereby higher-harmonic generation in a nanoisland sublattice drives a new, frequency-doubled mode in the other nanoisland sublattice. This situation is similar to that encountered in thin films, whereby backward volume waves scatter into surface waves^52^. Because the two nanoisland sublattices in a square ice are geometrically aligned at 90 degrees, this mechanism suggests that orthogonal spin-wave propagation can be achieved in these ASIs. Consequently, we open opportunities for the manipulation of magnon population and transport through geometric design^10,15^.

## Results

### Excitation of magnons in square ice

A multi-step lithography process is used to fabricate square ASI made of 20-nm-thick Ni_81_Fe_19_ directly on top of a coplanar waveguide (see Methods and Supplementary Figure 1). Individual elements have nominal lateral dimensions 260 × 80 nm^2^ and are separated by a 34-nm gap. The lattice constant defined as the center-to-center distance neighboring nanomagnets belonging to the same sublattice is 340 nm. Scanning electron microscopy images are shown in Supplementary Figure 1. The size discrepancy between the design and the fabricated nanoelements, as well as variability between elements, is smaller than 2 nm.

**Fig. 1:**
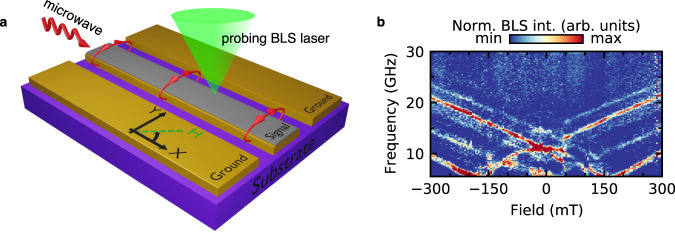
Experimental setup. **a** Combined microwave spectroscopy and microfocused Brillouin light scattering (BLS) measurement system. The square artificial spin ice (the gray area) is patterned directly onto the signal line of a coplanar waveguide (CPW). **b** Thermal magnon spectrum probed using BLS. No microwave signal is applied to the CPW.

Spin dynamics are detected by thermally excited and microwave-driven microfocused Brillouin light scattering spectroscopy (BLS); see Methods. A sketch of the measurement configuration is shown in Fig. 1a. The BLS process is the inelastic scattering of laser photons with magnons^53,54^.

**Fig. 2:**
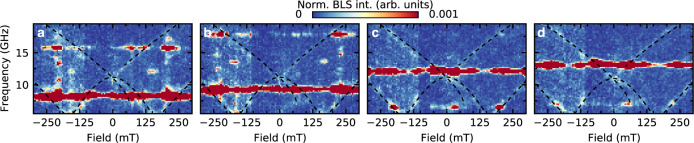
Measured spectra under microwave excitation. Driving microwave frequency-dependent magnon spectra in the nonlinear regime at nominal *P*_applied_ = 2 W (+33 dBm) and microwave frequencies of **a** 7.25 GHz, **b** 8.25 GHz, **c** 11 GHz, and **d** 12 GHz. The intense nearly-field independent band corresponds to the directly excited frequency. However, if there are states available at 2*f* or *f*/2 [compare to the thermal spectrum shown in Fig. 1b, with the main modes superimposed as black dashed lines], magnon scattering is observed as indicated by an increased BLS intensity.

Two different BLS measurement modalities are employed: (1) detection of thermally excited magnons without any microwave excitation^42^ and (2) detection of high microwave power excited nonlinear magnons. We consider the *k* = 0 mode as a magnon, following standard usage, see, for example, ref. ^49,55^. For the first measurement modality, the laser is focused on the sample surface at a fixed position, and the inelastically scattered light is then analyzed while a biasing magnetic field is swept from negative to positive values. A typical thermal magnon spectrum for a square ice lattice, where the biasing field is applied along one of the principal axes, is shown in Fig. 1b. We use the same sample for the second measurement modality and maintain the focused laser spot onto the same position during the measurements. However, we supply a high microwave power of nominally up to 2 W (+33 dBm). We record a field-dependent BLS spectrum for each applied constant excitation frequency, see Fig. 2. To minimize heating effects, the laser power for all BLS measurements is <1.5 mW at the sample position.

In the following, we discuss microwave-excited experiments. The field-dependent spectra show excitations in the ASI at the microwave excitation frequency as well as at higher and lower frequencies. For instance, the excitation at the microwave frequency can be observed as a horizontal line of increased BLS intensity in Fig. 2a–d. In Fig. 2a, besides the excitation at a microwave signal of 8 GHz, we also observe a secondary excitation at 16 GHz for a limited field range (~±170 mT). As the frequency of the microwave increases, excitation at lower frequencies appear, as is apparent in Fig. 2c–d. The intensity is the highest when there is an available thermal mode at a given field-frequency combination, seen in the thermal spectrum, Fig. 1b. A video of the BLS-spectra as a function of microwave field is discussed in Supplementary Note 2 and shown in Supplementary Movie 1.

These additional resonances occur precisely at harmonic and half-harmonic frequencies with respect to microwave excitation. At higher microwave power, additional secondary modes are observed, which will be discussed below. This immediately points to either higher-order modes or parametric pumping^56–58^. However, in our nanopatterned material, the magnon scattering processes cannot be described within the context of a dispersion continuum^59^ but rather in discrete modes that do not necessarily overlap in space. Therefore, insights into the observed processes must be obtained by studying the power dependence of these modes.

**Fig. 3:**
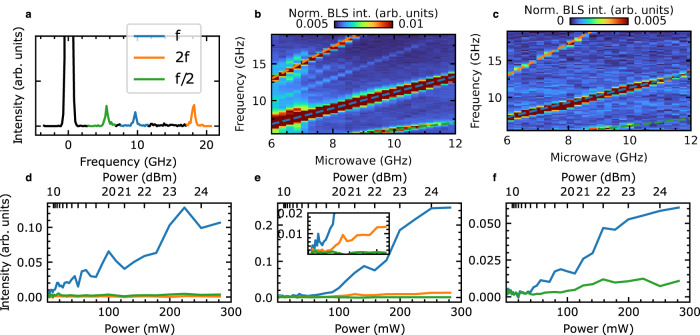
Experimental results. **a** Characteristic BLS spectrum showing the three excitations at *f*/2 (green), *f* (blue), and 2*f* (orange), at a microwave power of 0.3 W (25 dBm), external magnetic field of 100 mT and microwave frequency of 8.25 GHz. Note that the black peak centered at 0 GHz corresponds to the elastically scattered Rayleigh peak. Excitation modes as a function of microwave frequency at nominal powers of **b** 2 W (+33 dBm) and **c** 0.3 W (+25 dBm). The direct excitation at the microwave frequency *f* is shown with a blue dashed line. The 2*f* and *f*/2 excitation bands can also be observed and are shown with an orange or green dashed line, respectively. At the very high powers used in the measurements in **b**, other secondary modes can be observed. These modes are fractional modes due to three-magnon processes, i.e., *f*_frac_ = *f* + *f*/2 (faint mode observed between 2*f* and *f*). The slight broadening of the *f* and 2*f* signals may be due to four-magnon processes^74^. These secondary modes are not detected at a modest mircowave power of 0.3 W, **b**. The intensity of the modes increases with power as shown in **d**–**f** for specific field/frequency combinations of **d** 25 mT and 8.25 GHz, **e** 100 mT and 5.25 GHz, and **f** 100 mT and 10 GHz.

In Fig. 3a, we show a characteristic BLS spectrum showing three clear resonances for a bias magnetic field of 100 mT and a 0.3 W (+25 dBm) amplitude microwave excitation at 8.25 GHz. Here, we color-code the directly excited mode in blue, the second harmonic in orange, and the half-frequency in green. By integrating the full measured spectrum for each microwave frequency, we obtain the representation shown in Fig. 3b, c. We find that the integrated response qualitatively agrees with that of the continuum magnon process^60^, where we recognize the fundamental frequency *f* (blue dashed line), second harmonic 2*f* (orange dashed line), and half harmonic *f*/2 (green dashed line). In Fig. 3b, the nominal microwave power is 2 W (+33 dBm), leading to clean harmonics and even evidence of a fractional harmonic at 3*f*/2, which is a result of three-magnon scattering where one magnon at half harmonic *f*/2 and one magnon at the fundamental frequency *f* confluence to one magnon at *f*/2 + *f* = 3*f*/2. In Fig. 3c, the nominal microwave power is 0.3 W (+25 dBm). Despite the lower signal-to-noise, the harmonics are still visible.

The onset of the harmonics is clarified from the power-dependent amplitude of the modes, shown in Fig. 3d–f. When only the fundamental mode is excited, shown in panel d, we detect a finite intensity for any non-zero power. This is consistent with the resonance of a thermally excited mode. In contrast, when other modes are excited, there is a discernible threshold. We can understand this threshold as an induced excitation opposing magnetic damping^61^. Because our sample consists of nanoislands, the damping is further enhanced by non-uniform modes^62^. Within our experimental accuracy, we can fit the power threshold to 79 mW ± 3 mW^63^. The fits and procedure are discussed in the Supplementary Note 3. We note that the power dependencies observed in panels d, e are representative of the qualitative behavior found for other frequency and field combinations. However, the quantitative power difference between the excited modes does depend on the particular frequency and field combination.

### Numerical modeling

**Fig. 4:**
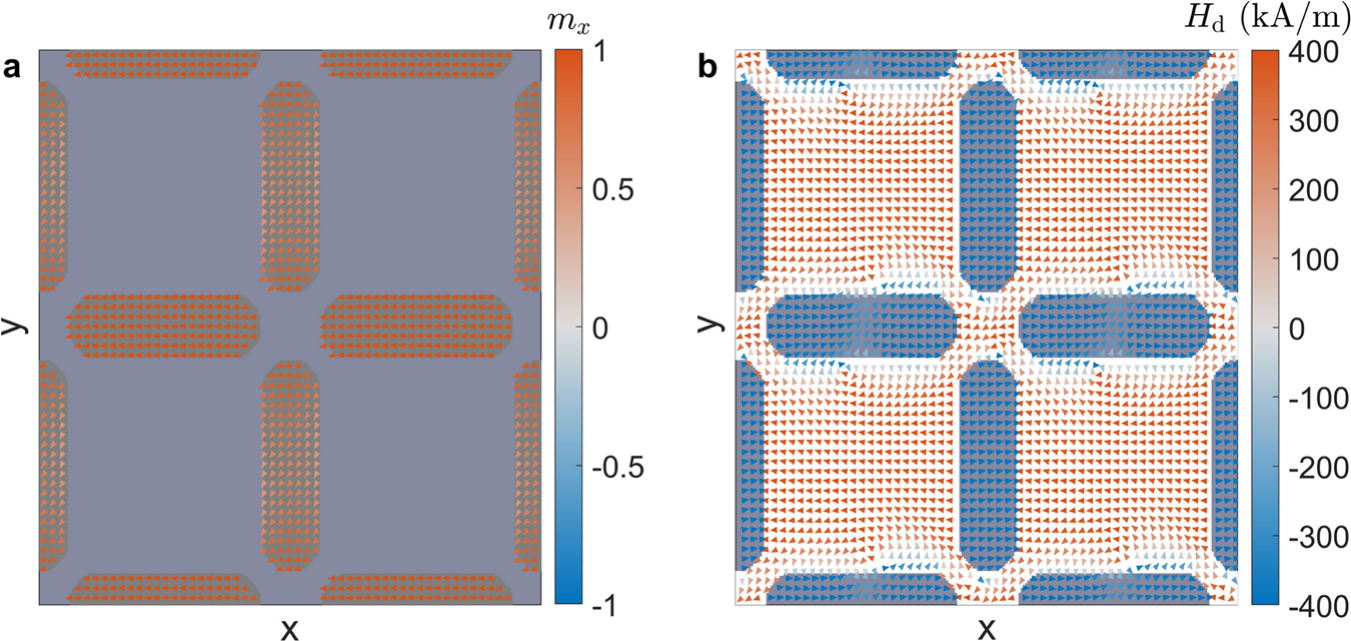
Static micromagnetic configuration under applied field. **a** Magnetization state under an applied field of *B*_ext_ = − 100 mT. **b** Computed magnetostatic field arising from the magnetic configuration shown in **a**.

We use micromagnetic modeling to understand how microwave power influences the onset of harmonic modes in ASIs (see Methods). To model the square ASI, we define a micromagnetic unit cell^10^ with periodic boundary conditions. We first minimize the energy of the structure to obtain the static magnetic state as a function of field. An example magnetic configuration is shown in Fig. 4a for the square ice unit cell under an external magnetic field of *B*_ext_ = − 100 mT along the *x* axis. This field condition was chosen based on the experimental results presented above. Under this field, the horizontal islands are fully magnetized and the magnetization is mostly uniform along the *x*-axis due to the nanoisland’s easy axis defined by shape anisotropy^64^. The vertical nanoislands are partly magnetized and exhibit substantial edge bending of the magnetization^65^. At the vertex, nanoislands can couple magnetostatically, as shown in Fig. 4b.

**Fig. 5:**
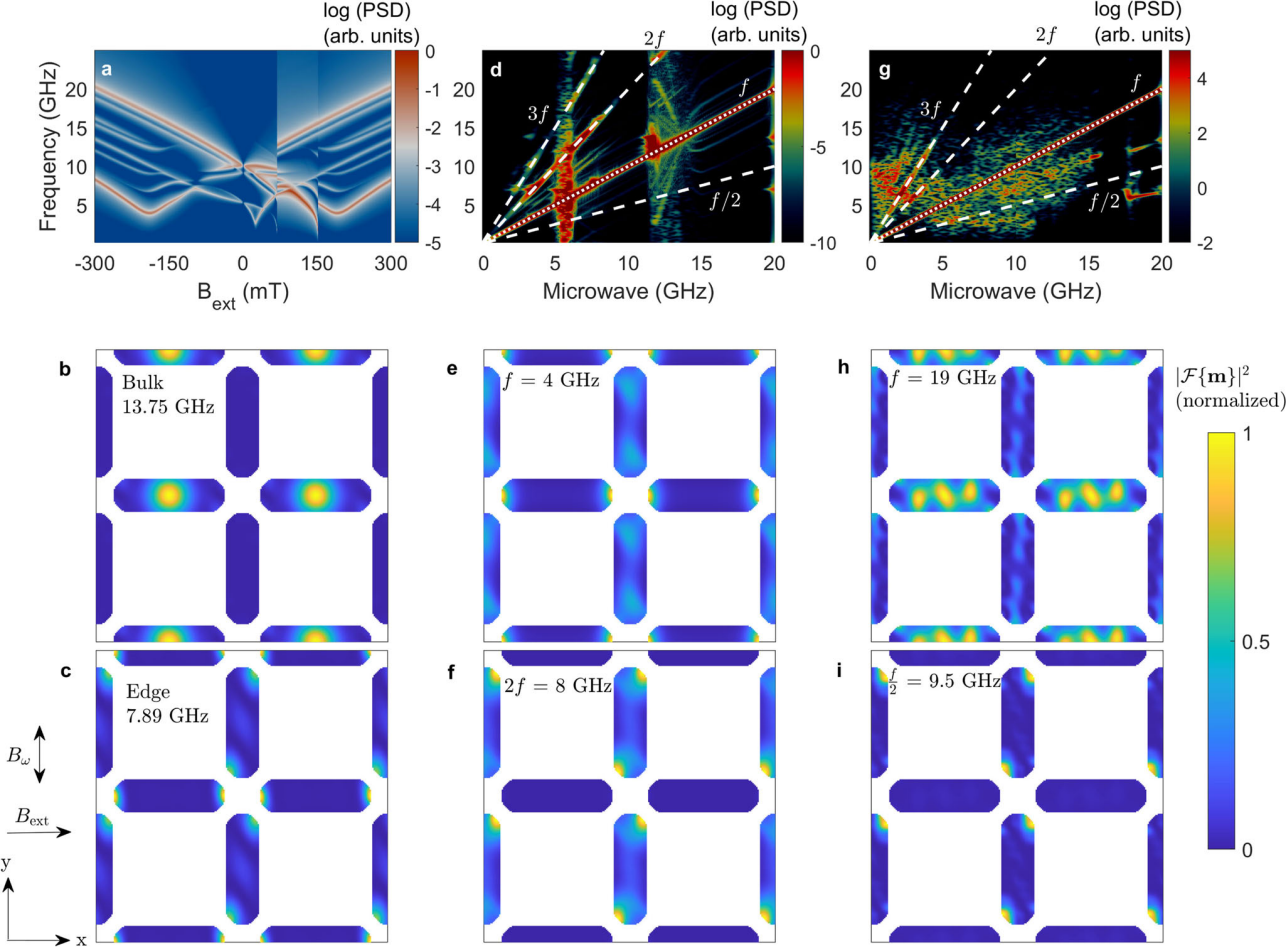
Micromagnetic simulations of square ASI. **a** Numerically simulated thermal modes for a square ice lattice. At *B*_ext_ = − 100 mT, the bulk and edge modes are shown in **b**, **c**, respectively. **d** Spectrum computed by a chirped microwave excitation of amplitude *B*_*ω*_ = 5 mT. The fundamental (*f*) and second harmonic (2*f*) modes for *f* = 4 GHz are shown in **e**, **f**, respectively. **g** Spectrum computed by a chirped microwave excitation of amplitude 50 mT. The fundamental (*f*) and half harmonic (*f*/2) modes for *f* = 19 GHz are shown in **h**, **i**, respectively. The orientation of the external and microwave fields is shown in the figure.

The magnon spectra of the system are shown in Fig. 5a, demonstrating good agreement with the thermal spectra obtained by BLS. It must be noted that the numerical magnon spectra are obtained by initializing the system in the fully magnetized direction with *B*_ext_ = − 300 mT and subsequently utilizing increasing field magnitudes until *B*_ext_ = + 300 mT. As such, the sharp spectral jump observed at ≈70 mT represents the reversal of the nanoislands parallel to the external field.

To study the spectra subject to a microwave excitation, we use a chirped microwave field so that its frequency increases linearly from 0 GHz to 20 GHz at a rate of 120 MHz/ns. The chirped microwave field allows us to numerically scan for the spectral features of the system in a numerically efficient way with the caveat that transient effects are also captured. A similar approach was used to study synchronization in spin-torque nano-oscillators^66^. The numerical analysis is performed using a smoothed pseudo-Wigner-Ville distribution (PSWV, see Methods and more details in Supplementary Note 4) previously used in the context of magnetization dynamics^28,67^. It is worth pointing out that the simulations capture only the *k* = 0 magnons, i.e., magnons at the Γ point.

We focus here on the spectra obtained in a uniform bias magnetic field *B*_ext_ = − 100 mT applied along the *x* direction. The system’s eigenvalues can be directly extracted from Fig. 5a, where we identify a dominant bulk mode at 13.75 GHz and an edge mode at 7.89 GHz. The spatial profiles of these modes are shown in Fig. 5b, c, respectively. The corresponding simulations for each ASI sublattice^68,69^ are discussed in Supplementary Note 5. The color plot represents the normalized sum of the Fourier amplitude for all magnetization components in each computational cell. Because the microwave field is applied along the *y*-direction it most efficiently couples to the horizontal nanoislands for the bulk mode. The excited edge modes are strongly coupled between horizontal and vertical nanoislands, as observed in panel c.

The spectra obtained by PSVW when the system is excited with a chirped microwave with amplitude *B*_*ω*_ = 5 mT is shown in Fig. 5d. This field magnitude is representative of a moderate microwave power excitation in experiments [compare to Fig. 3c]. The linear evolution of the resonance at the direct microwave frequency *f* is clearly observed and is emphasized by a dotted white line. In addition, we see evidence of resonances at harmonic branches, emphasized by dashed white lines. For example, strong spectral content is observed in the 2*f* branch between 3 GHz and 7 GHz and a weak content in the 3*f* branch between 5 GHz and 7 GHz. Interestingly, no discernible spectral content is observed in the *f*/2 branch. These simulations confirm that higher harmonics can be excited when in resonance with the thermal modes.

For example, we show the spatial mode distribution for the directly excited mode *f* = 4 GHz in Fig. 5e. Low-frequency edge modes are excited in the horizontal nanoislands and low-amplitude oblique modes in the vertical nanoislands. The oblique modes are a consequence of the partial saturation of the nanoislands due to the applied field, shown in Fig. 4a. In contrast, the 2*f* = 8 GHz frequency shown in Fig. 5f is primarily composed of oblique edge modes in the vertical nanoislands. This means that the 2*f* branch is a distinct edge mode that appears in vertical nanoislands due to their coupling to the horizontal nanoislands. The fact that the mode has twice the microwave frequency is indicative that the process is akin to frequency doubling, whereby the horizontal nanoislands act as nanoantennas that excite the vertical nanoislands edges at 2*f*. This is confirmed by the ellipticity of the horizontal mode, shown in Supplementary Note 6.

When the microwave field amplitude is increased to *B*_*ω*_ = 50 mT—corresponding to the strong excitation regime in our micromagnetic model – qualitatively distinct features are observed in the chirped spectra of Fig. 5g. From the broad distribution, it is immediately apparent that the low-frequency excitation leads to dynamics that cannot stabilize during the chirp. However, we see evidence of resonances at higher harmonics for microwave frequencies under 5 GHz. We emphasize 2*f* and 3*f* with dashed white lines. At higher frequencies, over ≈17 GHz, we observe indications of 2/3*f* and 1/3*f* modes, seen as straight lines around the *f*/2 dashed white line. At *f* = 19 GHz, the sample is excited well above the maximum thermal mode frequency obtained by simulations. Therefore, the fundamental mode profile corresponds to a higher-order bulk mode with a preferential location within the horizontal nanoislands, as shown in Fig. 5h. The half-frequency mode *f*/2 = 9.5 GHz, shown in panel g, is clearly an edge mode within the vertical nanoislands, which are parametrically pumped by the microwave excitation.

Further insights into the energy required to excite these modes can be obtained by quantifying the mode volume. To this effect, we integrate the mode profiles in each case to obtain a normalized power density. We take the value obtained for the thermal modes as a reference. The plotted Fourier power is normalized for each mode so that a one-to-one comparison can be obtained between thermal modes and microwave-excited modes, despite their different energy scales. We find a power density normalized to the thermal power density of 0.91 for the 2*f* mode at 8 GHz and 0.40 for the *f*/2 mode at 9.5 GHz. The fact that the energy density for the 2*f* mode is more than twice that of the *f*/2 is further evidence that it is excited by a different mechanism. The 2*f* mode effectively possesses all the energy of the thermal mode, indicating that it occupies the same volume. In contrast, the *f*/2 mode possesses barely half the energy density of the thermal mode, indicating that it coexists with another mode in the nanoisland. We interpret this as a demonstration that the *f*/2 mode is parametrically pumped as it shares the nanoisland volume with the directly excited mode.

### Comparison to other arrangements of nanomagnets

**Fig. 6:**
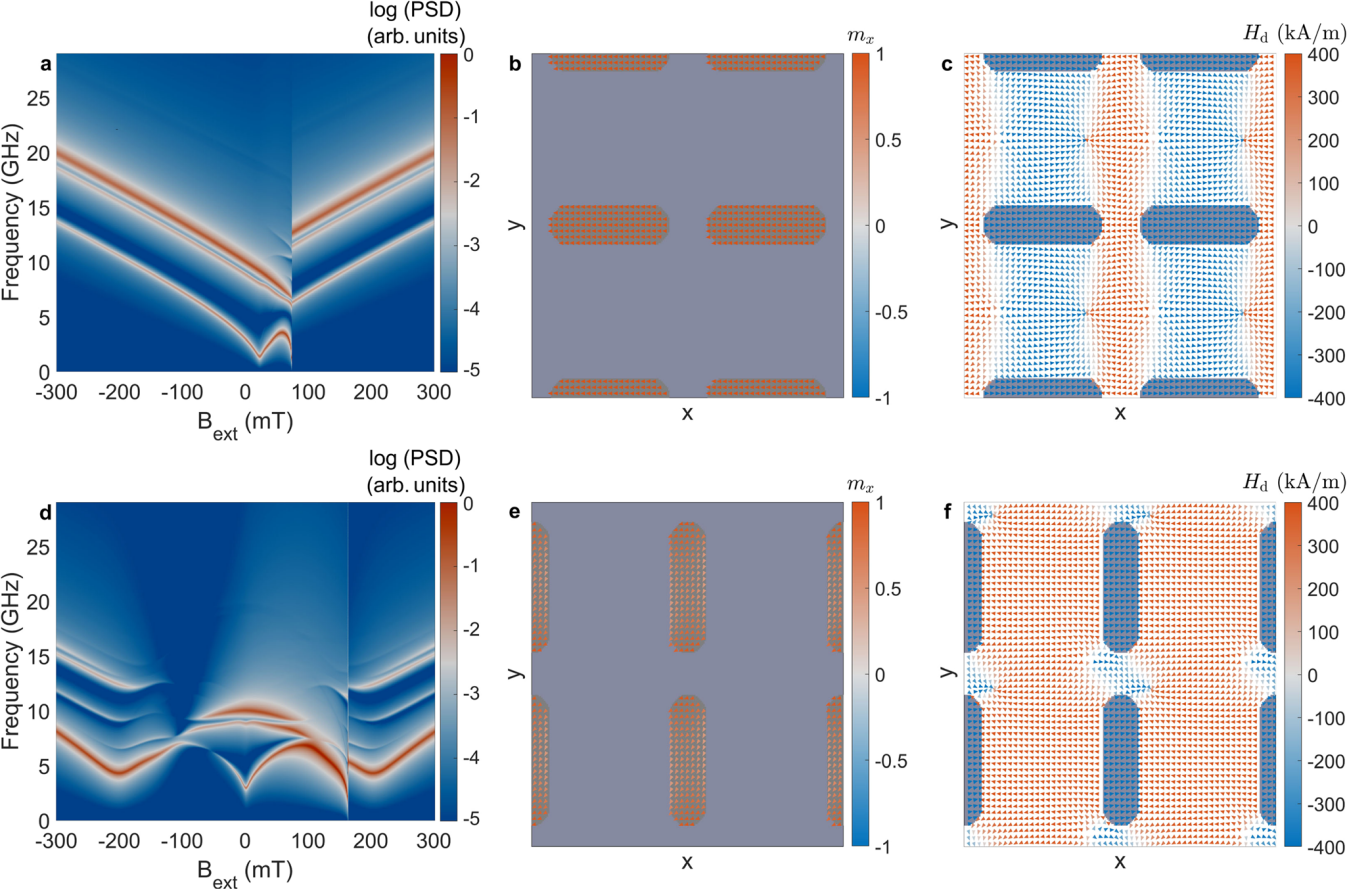
Micromagnetic simulations of individual ASI sublattices. **a** Thermal modes, **b** magnetization state, and **c** magnetostatic field for an ASI sublattice of horizontal nanoislands. **d** Thermal modes, **e** magnetization state, and **f** magnetostatic field for an ASI sublattice of vertical nanoislands.

To further investigate whether the 2*f* excitation of vertical nanoislands is unique to ASIs, we have performed simulations of individual ASI sublattices. In Fig. 6a, we show the thermal modes of an array of horizontal nanoislands, corresponding to the lowest-energy branches in Fig. 5a. The magnetization state when subject to an external field of −100 mT is shown in Fig. 6b. Similar to Fig. 4a, the nanoislands are fully magnetized as the field is oriented along the nanoisland’s easy axis. The magnetostatic field is shown in Fig. 6c, displaying coupling along the nanoisland’s chains. For an array of vertical nanoislands, the thermal modes, static magnetization, and magnetostatic field are shown in Fig. 6d, f. In this case, we also observe evidence of the branches in the ASI corresponding to the vertical nanoislands and a static magnetization exhibiting significant edge bending at −100 mT. The bulk and edge mode for each ASI sublattice agree with those shown is Fig. 5b, c, as expected from previous investigations, e.g., ref. ^37^. The simulation results for these modes are shown in Supplementary Fig. 3.

**Fig. 7:**
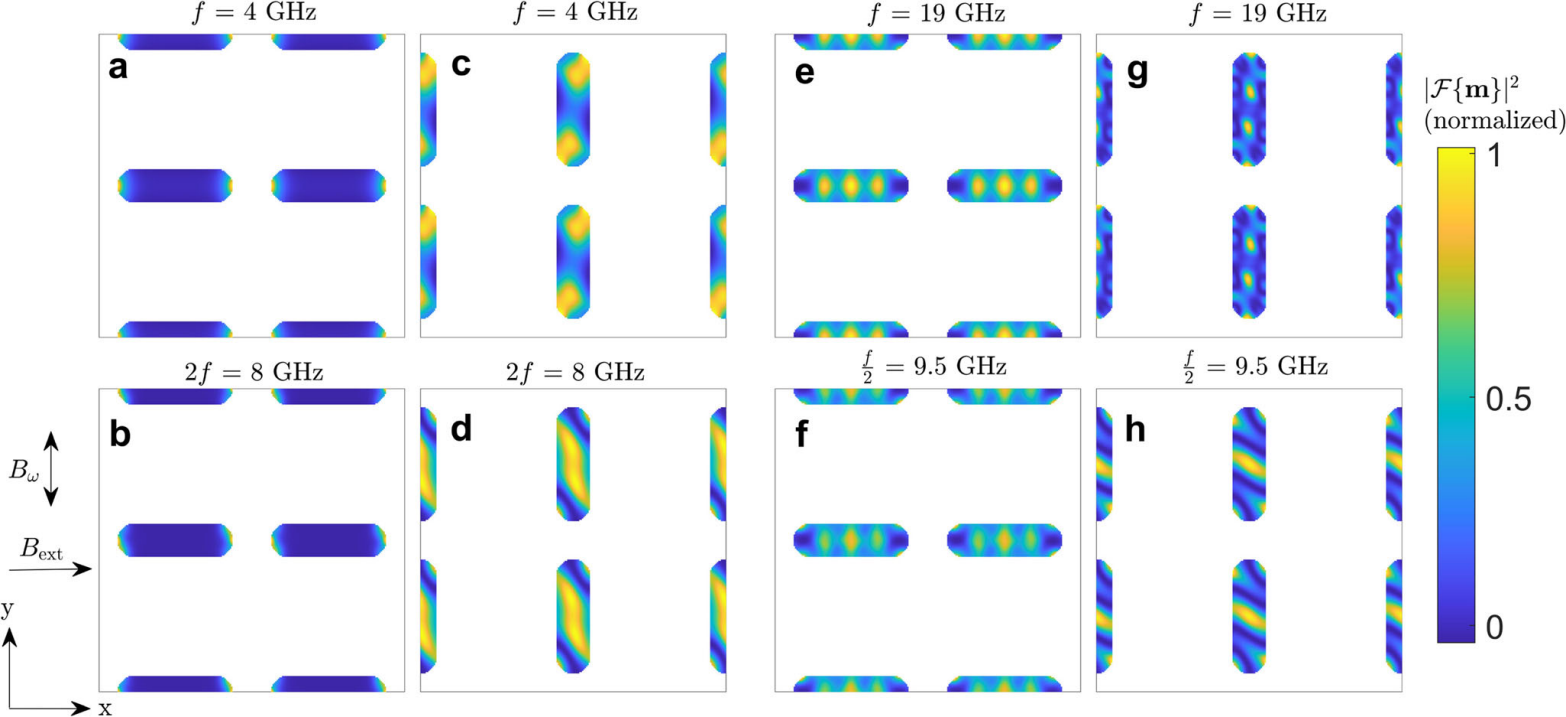
Modes in individual ASI sublattices under microwave excitation. The directly excited and second harmonic modes for a microwave excitation of *f* = 4 GHz and *B*_*ω*_ = 5 mT are shown, respectively, for the horizontal (**a**, **b**) and vertical (**c**, **d**) ASI sublattice. The directly excited and half harmonic modes for a microwave excitation of *f* = 19 GHz and *B*_*ω*_ = 50 mT are shown, respectively, for the horizontal (**e**, **f**) and vertical (**g**, **h**) sublattice ASI.

We then investigated the mode profile of each subset of ASIs under external microwave excitation. For 4 GHz at *B*_*ω*_ = 5 mT the mode profiles are shown in Fig. 7 and are to be compared with the modes of the full ASI in Fig. 5. The 4 GHz mode can be recognized in each of the sublattices and the full ASI, indicating that its profile is primarily due to the internal field within nanoislands (further simulations on single nanoislands are presented in the Supplementary Note 7). However, we observe a bulk mode at 8 GHz that is different than the primary edge mode at the same frequency in the ASI.

When these ASI sublattices are excited with a 19 GHz microwave at *B*_*ω*_ = 50 mT, we recover modes similar to those observed in the full ASI. The half-frequency mode excited in vertical islands has the same mode profile in both the full ASI and vertical sublattice, Figs. 5i and Fig. 7h, confirming that these mode are excited by parametric pumping. In the case of the horizontal nanoislands, Figs. 5h and  7e show similar mode volumes, except from spatial distortions attributed to differences in the magnetostatic field.

## Discussion

Our experimental and numerical results demonstrate that harmonic magnon modes can be excited in ASIs subject to sufficiently strong microwave power. This excitation induces a spatial modification of modes and, consequently, enables nonlinear processes in the ASI. In particular, we find a unique mechanism in ASI where higher-harmonic generation in a nanoisland subset induces a distinct frequency-doubled mode in the other subset of nanoislands. This feature is unique to nanostructures with dissimilar sublattice properties. Despite the discrete nature of these modes, as shown in Figs. 1b and  5a, we find that the field-dependent behavior of the magnon spectrum exhibits similarities to its continuum counterpart^60^.

It is essential to emphasize that the results presented here carry limited information on the propagation direction of the modes. Experimentally, we detect a range of wavevectors while simulations return uniform modes. The good agreement between experiments and simulations suggests that the spectrum observed here is primarily due to **k** = 0 waves. However, it is clear that those waves are located in particular sublattices of the square ASI. This analysis implies that energy can be distributed in orthogonal modes, which, for finite wavevectors, further leads to the possibility of orthogonal spin-wave channels. From the point of view of applications, orthogonality implies minimal cross-talk between information sent simultaneously through different modes. The uncovered nonlinear magnon scattering in the studied square ASI opens the possibility of energy transfer dictated by geometry. We expect that this mechanism could be employed as a power-dependent nonlinear fading memory with distinct sublattice responses that could be leveraged for magnon reservoir computing^40^.

Numerical simulations indicate that the magnon scattering process observed here is made possible by frequency doubling mediated by the nanoislands that are directly excited, as long as a mode volume is available. We argue that the discrete spectrum of ASI at a given field condition can be considered to have a finite linewidth that defines a “bandwidth” for magnon scattering. This opens fascinating opportunities to manipulate magnons for information technologies based on nanoscale patterned functional magnetic materials. The excitation of magnons through different sublattices allows one to envision the use of bi-component ASI^37^ and hybrid ASI^28,70^ to selectively excite single or multiple magnon channels carrying information by angular momentum. By reconfiguring the magnetic state of ASIs, it would also be possible to manipulate how information is carried in space. For example, a hybrid device can be envisioned where spin waves in an extended film can be selectively steered by dipolar coupling to an ASI patterned on top of it. Extensions to other types of ASI geometries could also be possible, e.g., a charge ice^71^ that is easily reconfigured by external fields.

We, therefore, believe that the presented results will open new avenues to manipulate the magnon populations in reconfigurable ASIs and initiate research on tunable and co-existing magnon transport at different frequencies in a single functional material.

## Methods

### Sample fabrication

A multi-step lithography process was used for sample fabrication. We first defined a coplanar waveguide (CPW) and alignment marks on a thermally oxidized Si substrate using electron-beam lithography, electron-beam evaporation of 10-nm Ti and 150-nm Au followed by lift-off. The width of the signal line is 20 μm, separated by an 8 μm gap between the signal and the 40 μm wide ground lines. The total length of the CPW is 1.5 mm. The ASI structure was written directly on the signal line by electron-beam lithography. For this purpose, a double-layer positive resist of methyl methacrylate/polymethyl methacrylate (MMA/PMMA) was used. After exposure and development of the resist stack, we used electron-beam evaporation and deposited 5 nm Ti, 20-nm Ni_81_Fe_19_, and 5 nm Ti capping layer, followed by a lift-off step. Individual ASI elements have lateral dimensions 260 × 80 nm^2^ and are separated by a 34-nm gap.

### Brillouin light scattering spectroscopy

Microfocused Brillouin light scattering spectroscopy measurements are performed in a back-scattering geometry using a continuous wavelength single-mode 532 nm wavelength laser. We use a high-numerical-aperture (NA = 0.75) objective lens with a magnification of 100×, which collimates the scattered and reflected light within a large cone angle with respect to the sample surface normal. Hence, an extensive wavevector range between 0 and 17.8 rad μm^−1^ is detected. The optical resolution of the system is less than ≈ 500 nm. A light source is used to illuminate the sample for monitoring the measurement position. An auto-focus routine based on the readout of the reflected laser intensity by a photodiode is used. The inelastically scattered light is analyzed using a high-contrast multi-pass tandem Fabry Pérot interferometer with a contrast of at least 10^15^. The magnon signal is extracted from the Stokes peak. All BLS measurements are conducted at room temperature. The acquisition time for the thermal magnon spectrum was set to about 10 minutes per BLS spectrum for one field value. Each BLS spectrum was scanned from −30 GHz to 5.5 GHz. For the excitation spectra, the acquisition time was reduced to 15 s per BLS spectrum for one field value. In this case, each BLS spectrum was scanned from −20.65 GHz to 3.5 GHz. For the microwave-excited measurements, a microwave signal from a BNC845 microwave source is amplified by a broadband microwave amplifier (PE15A4001).

### Micromagnetic simulations

Micromagnetic simulations were performed using the open-source GPU accelerated package MuMax3^72^. The lateral nanoisland dimensions and lattice parameters are taken from the nominal experimental dimensions. The simulated unit cell is a domain of 230 × 230 × 1 cells, with cells of dimensions 2.97 nm × 2.97 nm × 15 nm. We use magnetization parameters for bulk permalloy: saturation magnetization *M*_*s*_ = 800 kA m^−1^, exchange constant *A* = 13 pJ m^−1^, and Gilbert damping parameter *α* = 0.01. We have verified that we recover the same qualitative results for the thermal modes and at least one microwave field excitation with much smaller cell size in a 3D simulation, see Supplementary Note 8.

The magnetization state for each field is obtained by minimizing the energy with MuMax3 built-in relaxation function. The simulation is run for 5 ns after the energy minimization to avoid spin waves due to the relaxation of the magnetization^28^.

The thermal spectrum is obtained by exciting the relaxed magnetic state with a homogeneous external field applied along the *y* direction that has a sinc temporal profile. The field amplitude is set to 0.1 mT, and the sinc bandwidth is 50 GHz. In other words, this sinc field excites all the frequencies with the same amplitude up to a cut-off of 50 GHz. Therefore, this excitation mimics the excitation of thermal modes, without the numerical overhead of using a randomly fluctuating field. The data is sampled at 10 ps and run for 10 ns, achieving a spectral resolution of 100 MHz.

The chirped spectrum is obtained by a combination of two simulations due to limitations in the data processing of the smoothed Pseudo-Wigner-Ville (SPWV) distribution. For each simulation, the system is led out of equilibrium by a 10 ps field pulse of 0.1 mT applied at 45 degrees from the *x* axis. After this pulse, the system is excited with a chirp at a rate of 120 MHz ns^−1^. The simulation time is 100 ns, sampled at 10 ps. We run the chirp frequency from 0 GHz to 12 GHz and from 8 GHz to 20 GHz. The SPWV routine is implemented for each simulation result. Both simulations are then merged at 10 GHz, using a 2 GHz buffer to account for numerical errors from the SPWV calculation. The outcome is a seamless response from both chirps.

### Smoothed Pseudo-Wigner-Ville algorithm

The SPWV distribution allows one to obtain a time-frequency representation of a time trace. In contrast to other common implementations, e.g., sliding FFT, the Wigner-Ville transform precisely identifies the instantaneous frequency at a given instant of time. However, this transformation has known artifacts, so it is commonly smoothed in both the time and frequency domain. A typical implementation uses a Gaussian window for the time domain and a Hann window for the frequency domain. A good resource for the implementation can be found in Ref. ^73^. Here, we use an in-house implementation developed by one of the authors, used in previous publications^28,67^.

Here, the temporal window is related to the chirp frequency. We use a Gaussian window equivalent to 1 mT ns^−1^. A Gaussian window smoothes the frequency with *σ* = 0.2 GHz.

## Supplementary information

Supplementary Information

Description of Additional Supplementary Files

Supplementary Movie 1

## Data Availability

The raw and processed data that support the findings of this study are available from 10.17605/OSF.IO/YUNHD.

## Code availability

Computer codes for the micromagnetic simulations in mumax3 are available from 10.17605/OSF.IO/YUNHD.
