# Supplementary material for: Nonlinear multi-magnon scattering in artificial spin ice
Source: Nat Commun. 2023 Jun 9;14:3419. doi: 10.1038/s41467-023-38992-7 (PMC10256710; doi:10.1038/s41467-023-38992-7)
Supplement: Supplementary file 1 — Supplementary Information [file 41467_2023_38992_MOESM1_ESM.pdf]

Supplementary Information: Nonlinear multi-magnon scattering in artificial spin ice

Sergi Lendinez,[1,2] Mojtaba T. Kaffash,[1] Olle G. Heinonen,[3,*]
Sebastian Gliga,[4] Ezio Iacocca,[5,6,†] and M. Benjamin Jungfleisch[1,‡]

[1]*Department of Physics and Astronomy, University of Delaware, Newark, DE 19716, USA*

[2]*Center for Advanced Microstructures and Devices, Louisiana State University, Baton Rouge, LA 70806, USA*

[3]*Materials Science Division, Argonne National Laboratory, Lemont, Illinois 60439, USA*

[4]*Swiss Light Source, Paul Scherrer Institute, 5232 Villigen PSI, Switzerland*

[5]*Department of Mathematics, Physics, and Electrical Engineering, Northumbria University, Newcastle upon Tyne NE1 8ST, United Kingdom*

[6]*Center for Magnetism and Magnetic Nanostructures, University of Colorado Colorado Springs, Colorado Springs, CO 80918, USA*

[*] Present and permanent address: Seagate Technology, 7801 Computer Ave., Bloomington, MN 55435

[†] eiacocca@uccs.edu

[‡] mbj@udel.edu

SUPPLEMENTARY NOTE 1. SCANNING ELECTRON MICROSCOPY

Supplementary Figure 1 shows scanning electron microscopy images of the ASI under study. The size discrepancy between the design and the fabricated nanoelements, as well as variability between elements, is smaller than 2 nm.

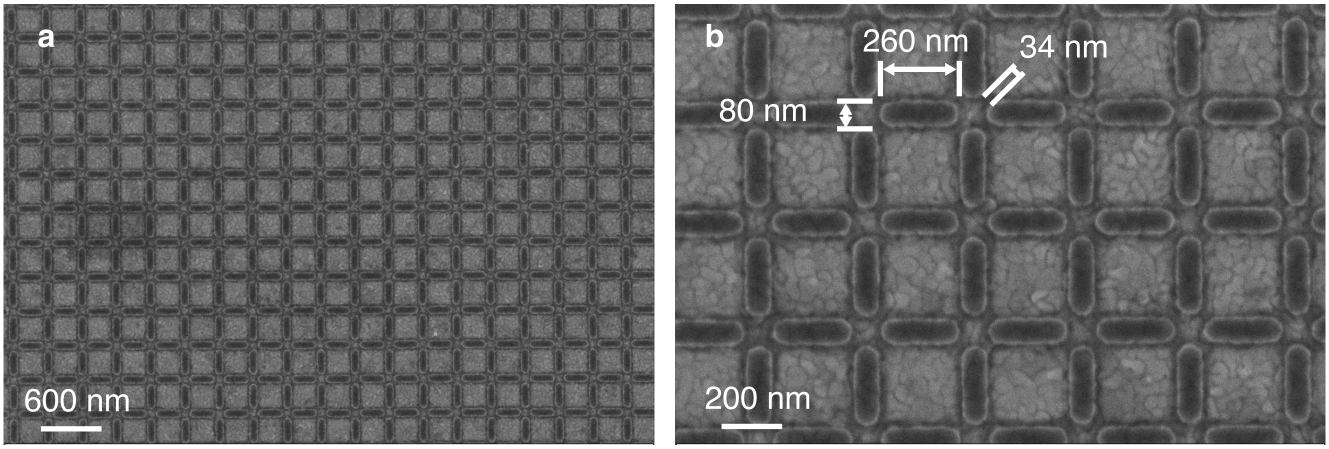

Supplementary Fig. 1. Scanning electron microscope image of the ASI under study at magnifications of **a** $86.72{\cdot}10^3\times$ and **b** $275{\cdot}10^3\times$.

SUPPLEMENTARY NOTE 2. MICROWAVE FREQUENCY SWEEP

A video showing the BLS intensity in a frequency vs. magnetic field plot while sweeping the excitation frequency from 5 GHz to 18 GHz at a fixed nominal power of 2 W (+33 dBm) is shown in the Supplementary Movie 1.

SUPPLEMENTARY NOTE 3. POWER THRESHOLD FITS

We approximate the threshold behavior using the auto-oscillator theory [1, 2] to estimate the threshold power. The theory predicts that a generalized auto-oscillator has a power described by

$$P \propto 1 - \frac{1}{\xi}, \tag{1}$$

where $\xi = P_\omega / P_{\mathrm{th}}$ is called the supercriticallity parameter and it is a ratio of the microwave power to the microwave threshold power. Because this equation predicts an output power

that increases asymptotically, it is difficult to fit a threshold, especially for experimental data with noise. A better representation consists in plotting the microwave power,

$$\frac{P_{\text{th}}}{P_\omega} \propto 1 - P, \tag{2}$$

so that the resulting data can be fitted with a line. We found that for this data set, smaller error bars in the fitted quantities were obtained by setting zero as the noise-floor, $< P >$.

A particular difficulty with our data set is the lack of points after the threshold is detected. For this reason, we consider an increasing number of data points and determine the threshold based on the best fit. An example of the best fit is shown in Supplementary Fig. 2. The threshold is then determined as the point where the fit crosses $< P > -P = 0$. We obtain a threshold power of 100 mW ± 3 mW (20 dBm ± 5 dBm) from this fit.

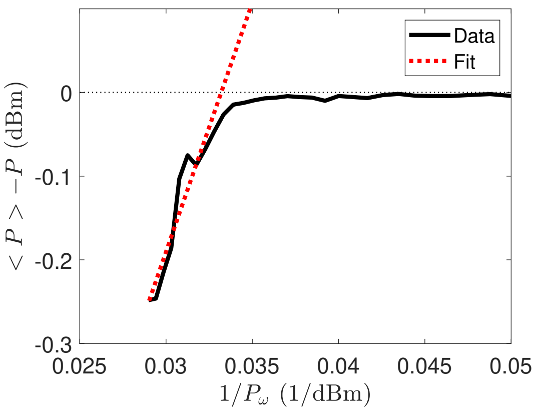

Supplementary Fig. 2. Example of a linear fit of the data plotted according to Eq. (2) and where $< P >$ indicates that the noise-floor is used to define zero (black dashed line).

SUPPLEMENTARY NOTE 4. CHIRPED SIMULATIONS

The main idea behind chirped micromagnetic simulations is to save computational time. Experimentally, the spectra are obtained by capturing a time-averaged spectrum for each experimental condition: bias field, microwave frequency, and microwave power. The signal-to-noise ratio is improved by averaging multiple spectra. This technique can be reproduced numerically but at a high computational cost. For example, suppose a microwave excitation at 1 GHz and a requirement to obtain a spectrum with a frequency resolution of 100 MHz,

comparable to BLS. According to the Nyquist criterion, the minimum sampling time will be 0.5 ns from the microwave frequency. However, the full spectrum up to 20 GHz, e.g., in Fig. 2, requires a sampling time of at least 25 ps, increasing the trace to 400 points. Considering that a single oscillation of the microwave frequency is insufficient to achieve a steady-state, a typical stabilization time of 10 ns must be considered. This already introduces an 11-ns-long simulation for each experimental condition. Finally, we note that the sampling time is different from the time step of the partial differential equation method solver used. This time step is typically an order of magnitude smaller, i.e., 1 ps. Overall, at least 11,000 iterations would be required per experimental condition. This argumentation illustrates the enormous computational resources required to reproduce Fig. 2 by simulating the experimental method.

An alternative solution is to use a smooth change in the external conditions to approximately recover the experimental results. This technique economizes the use of resources with acceptable accuracy, as clearly illustrated in Ref. [3]. Here, we introduce this technique for the case of microwave excitation in artificial spin ices, where the rate of the chirp was adjusted to minimize transient effects. We also perform the simulation in blocks due to the large amount of data required to compute the smoothed pseudo-Wigner-Ville distribution [4, 5]. As such, we can perform only two 400 ns long simulations to explore a range of 20 GHz of microwave excitations. Brute-force simulations replicating the BLS experimental approach would instead require a net simulated time of 18 μs, almost 46 times longer simulation time.

As mentioned above, this technique is prone to artifacts. An example is the broad spectrum in Fig. 5**d** at about 6 GHz. This feature appears because of a transition in the oscillations and thus the transient dynamics towards stabilization are captured as well. Despite these artifacts, most features are accurately described, allowing us to choose specific parameters to run dedicated simulations and resolve the magnetic mode volumes.

SUPPLEMENTARY NOTE 5. ASI SUBLATTICE MODES

We have previously shown that each branch in the thermal modes can be related to each sublattice, e.g., [6–8]. This is confirmed in the ASI sublattice mode simulations shown in Supplementary Fig. 3.

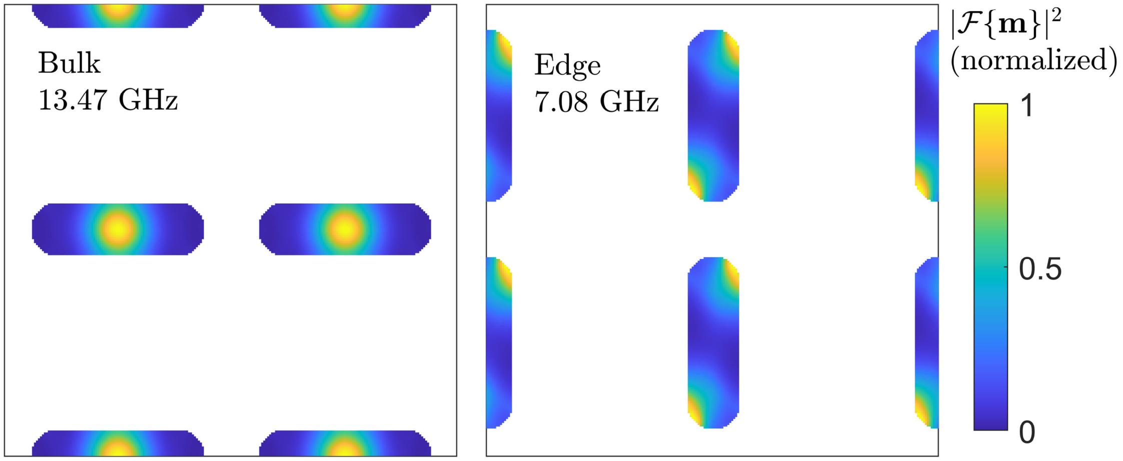

Supplementary Fig. 3. Modes of ASI sublattices. The dominant modes for the ASI sublattices correspond to **a** bulk in the horizontal sublattice anbd **b** edge in the vertical sublattice. These modes agree well with those shown in the main text, Fig. 5**b** and **c**. We note that the frequencies are slightly different, 0.3 GHz for the bulk mode and 0.8 GHz for the edge mode. This is due to the different field conditions in each ASI sublattice compared with the full ASI.

SUPPLEMENTARY NOTE 6. ELLIPTICITY OF 4 GHZ MODE AT HORIZONTAL NANOISLANDS

We have argued in the manuscript that parametric pumping/ higher harmonic generation are achieved in our experiments due to the strong microwave excitation. In addition, we have found that a frequency-doubled mode is excited in vertical nanoislands that are not due to higher harmonic generation, nor such modes are observed in ASI sublattices. The frequency-doubled mode is argued to appear as a consequence of higher harmonic generation in the horizontal nanoislands and these, in turn, acting as nanoantennas that excite the vertical nanoislands at the double frequency. Here, we show that indeed the horizontal nanoislands excite the vertical elements due to higher harmonic generation.

A tell-tale of higher harmonic generation is ellipticity in the magnetization precession since the magnetizaton orbit requires a nonzero component in its static direction to be feasible in the unit sphere. From the micromagnetic simulations presented in the main text, we can estimate the average magnetization trajectory at the edge of the horizontal

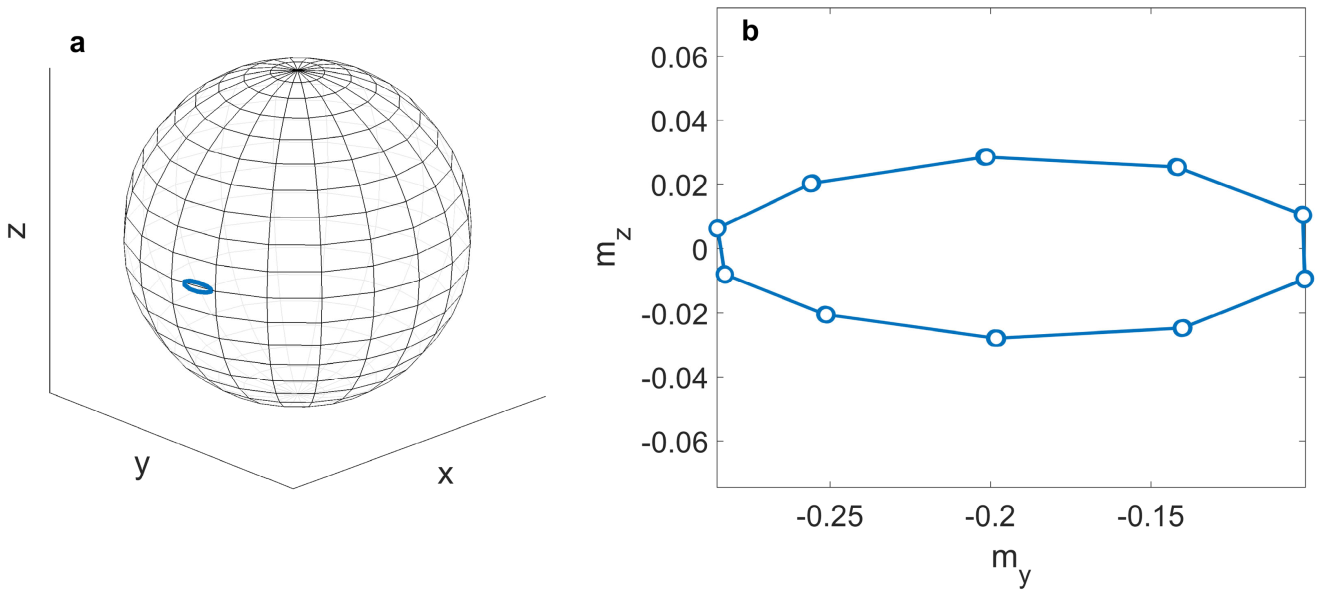

Supplementary Fig. 4. Ellipticity of edge modes in horizontal nanoislands excited at 4 GHz. **a** Average precessional orbit of the horizontal nanoisland in the unit sphere. **b** projection of the orbit in the y-z plane, clearly exhibiting an elliptic orbit.

nanoislands excited with 4 GHz at a field of $B_\omega = 5$ mT. To this end, we analyze the time-dependent, average magnetization of only 18 micromagnetic cells at the edge of a horizontal nanoisland. We then normalize the obtained magnetization and plot them in the unit sphere in Supplementary Fig. 4**a**. The small precession occurs about the m_x component since the horizontal islands are magnetized along that direction. It is apparent that the trajectory is elliptic. For clarity, we plot the projection of the magnetization trajectory in the $y-z$ plane in Supplementary Fig. 4**b**.

SUPPLEMENTARY NOTE 7. MICROWAVE EXCITATION OF SINGLE NANOISLANDS

Here, we perform simulations for single nanoislands oriented parallel and perpendicular to the external field to complement the results obtained for the full ASI and ASI sublattice presented in the main text. The goal of these simulations is to isolate dynamics present at the nanoisland level by direct microwave excitation. The magnetization states are shown in Supplementary Fig. 5**a**, in good agreement with magnetization states of nanoislands

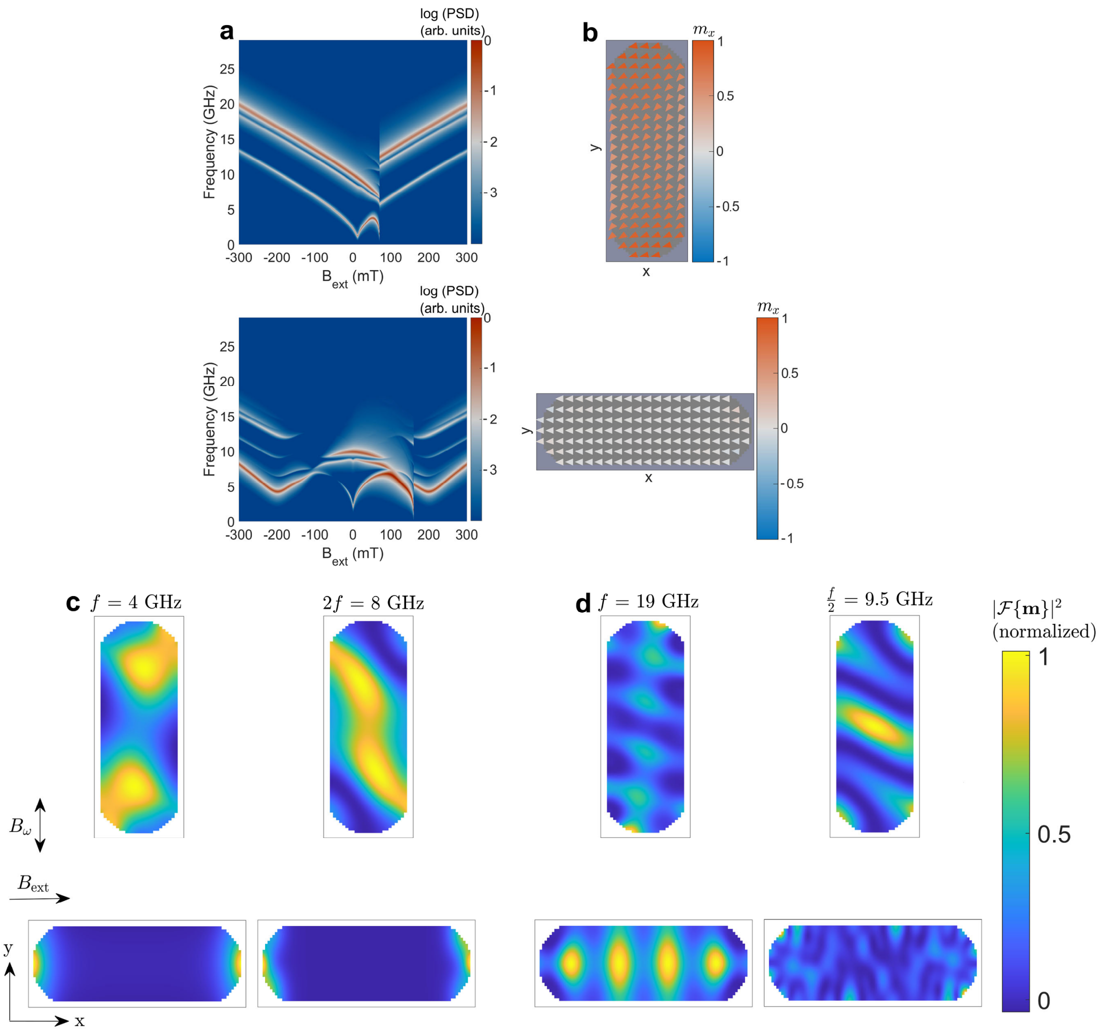

Supplementary Fig. 5. Micromagnetic simulation of microwave-excited nanoislands. **a** Thermal spin wave modes for individual nanoislands, showing the same characteristic features as the respective sublattice arrays, c.f. Fig. 6 in the main text. **b** Static magnetization state under an external field of -100 mT in the direction shown in the figure. **c** Directly excited and second harmonic modes for a microwave excitation of $f = 4$ GHz and $B_\omega = 5$ mT for both nanoislands. **d** Directly excited and half harmonic modes for a microwave excitation of $f = 19$ GHz and $B_\omega = 50$ mT for both nanoislands.

array. This demonstrates that the magnetic state is primarily determined by the internal energy, in particular shape anisotropy. The thermal spectrum as a function of field shown in Supplementary Fig. 5**b**, respectively, is also similar to those obtained for the horizontal and vertical sublattices. So far, these results indicate that the salient features of the magnon

spectrum are only slightly modified by the magnetostatic coupling between nanoislands. This is well known in the literature, and only edge modes are expected to interact between nanoislands [9].

The modes obtained under microwave excitation are shown in Fig. 5**c** for $f = 4$ GHz at $B_\omega = 5$ mT and Supplementary Fig. 5**d** for $f = 19$ GHz at $B_\omega = 50$ mT. Comparison with the results of ASI sublattices in the main text, Fig. 7, we observe essentially the same modes, barring minor mode profile distortion in the ASI sublattice due to the magnetostatic field. With these simulations, we further corroborate that parametric pumping occurs within the nanoislands and that the mode profiles are mainly determined by the internal field in the nanoislands.

SUPPLEMENTARY NOTE 8. 3D SIMULATIONS

To investigate the impact of our cell dimensions, we performed more detailed simulations using a reduced cell size of dimensions 1 nm $\times$1 nm $\times$1 nm. These cells have three times smaller lateral dimensions compared to those used in Fig. 5 and are also cubic, which improves the accuracy of the computation. The total number of cells for our geometry is $690 \times 690 \times 15$ resulting in ≈ 7.1 million cells. In contrast, the simulations presented in the main text require only ≈ 53 thousand cells. The significant increase in total cells results in a much longer simulation time. For example, a single microwave excitation to obtain mode profiles required 72 hours compared to 2 hours for the results presented in the main text.

In Supplementary Fig. 6, we present results from simulations with the reduced cell size for both the thermal modes and the case of a microwave excitation of $f = 4$ GHz and $B_\omega = 5$ mT. The mode profiles are shown in panels **a** and **b** for the m_y component. We observe a similar spatial distribution as in the main text Fig. 5**b** and **c**. However, we find a quantitative discrepancy in the modes' frequency. In particular, the edge mode is found at 8.08 GHz and the bulk mode at 15 GHz, cf. 7.89 GHz and 13.75 GHz, respectively, shown in Figure 5 in the main text. The discrepancy originates from a slightly different distribution of the magnetization state at that particular field. The nanomagnets find themselves in a preferentially horizontal configuration rather than a tilted configuration for the 3D simulations presented here. This is due to the rougher edges and lack of volume resolution in the results with larger cells.

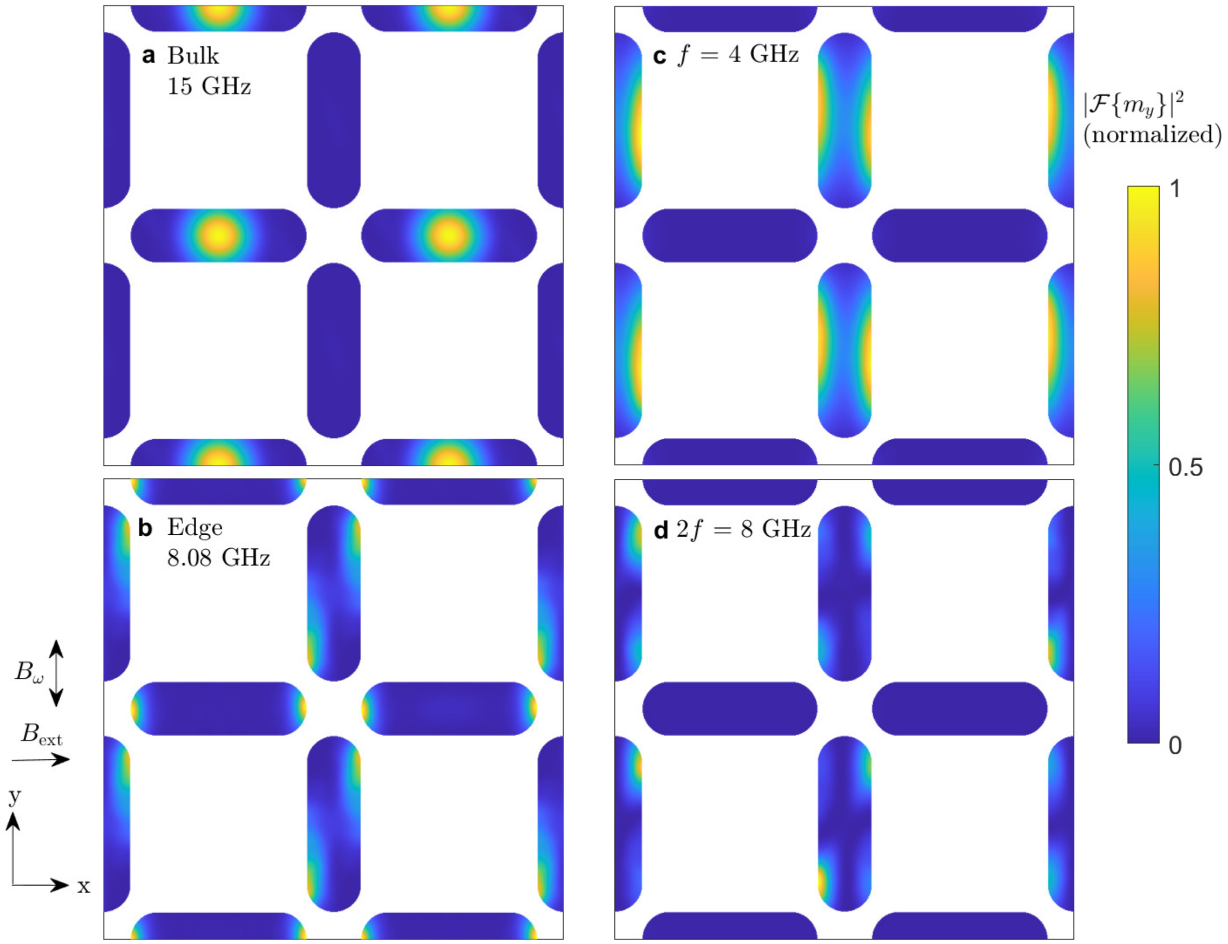

Supplementary Fig. 6. Spatial mode distributions of the m_y component using a reduced cell size of dimensions 0.989 nm $\times$0.989 nm $\times$1.0 nm. **a** bulk and **b** edge thermal modes at $B_{\text{ext}} = -100$ mT. **c** Directly excited mode by a microwave excitation of amplitude $B_\omega = 5$ mT and $f = 4$ GHz. **d** $2f$ mode excited by the microwave.

We then compute the modes obtained with a microwave field of $f = 4$ GHz and $B_\omega =$ 5 mT. The results are shown in panels **c** and **d**. Because of the different magnetization states, we find that the magnetization in the horizontal nanoislands is not excited in this simulation. Instead, the vertical nanoislands can more easily couple to the microwave field due to the reduced anisotropy in the y-direction. We find, nonetheless, a qualitative agreement in the excitation of the direct 4 GHz mode and the appearance of a $2f$ harmonic that is spatially located in a different section of the sample.

SUPPLEMENTARY REFERENCES

---

[1] A. Slavin and V. Tiberkevich, Excitation of spin waves by spin-polarized current in magnetic nano-structures, Magnetics, IEEE Transactions on **44**, 1916 (2008).

[2] A. Slavin and V. Tiberkevich, Nonlinear auto-oscillator theory of microwave generation by spin-polarized current, Magnetics, IEEE Transactions on **45**, 1875 (2009).

[3] T. Kendziorczyk, S. O. Demokritov, and T. Kuhn, Spin-wave-mediated mutual synchronization of spin-torque nano-oscillators: A micromagnetic study of multistable phase locking, Phys. Rev. B **90**, 054414 (2014).

[4] R. K. Dumas, E. Iacocca, S. Bonetti, S. R. Sani, S. M. Mohseni, A. Eklund, J. Persson, O. Heinonen, and J. Åkerman, Spin-wave-mode coexistence on the nanoscale: A consequence of the oersted-field-induced asymmetric energy landscape, Phys. Rev. Lett. **110**, 257202 (2013).

[5] E. Iacocca, S. Gliga, and O. G. Heinonen, Tailoring spin-wave channels in a reconfigurable artificial spin ice, Phys. Rev. Applied **13**, 044047 (2020).

[6] W. Bang, F. Montoncello, M. B. Jungfleisch, A. Hoffmann, L. Giovannini, and J. B. Ketterson, Angular-dependent spin dynamics of a triad of permalloy macrospins, Phys. Rev. B **99**, 014415 (2019).

[7] W. Bang, F. Montoncello, M. T. Kaffash, A. Hoffmann, J. B. Ketterson, and M. B. Jungfleisch, Ferromagnetic resonance spectra of permalloy nano-ellipses as building blocks for complex magnonic lattices, Journal of Applied Physics **126**, 203902 (2019), https://doi.org/10.1063/1.5126679.

[8] S. Lendinez, M. T. Kaffash, and M. B. Jumgfleisch, Emergent spin dynamics enabled by lattice interactions in a bicomponent artificial spin ice, Nano Lett. **21**, 1921 (2021).

[9] L. J. Heyderman and R. L. Stamps, Artificial ferroic systems: Novel functionality from structure, interactions and dynamics, Journal of Physics: Condensed Matter **25**, 363201 (2013).
