# Supplementary material for: Nonlinear multi-magnon scattering in artificial spin ice
Source: Nat Commun. 2023 Jun 9;14:3419. doi: 10.1038/s41467-023-38992-7 (PMC10256710; doi:10.1038/s41467-023-38992-7)
Supplement: Supplementary file 2 — Description of Additional Supplementary Files [file 41467_2023_38992_MOESM2_ESM.pdf]

Title: Supplementary Movie 1

Description: Measured Brillouin light scattering spectra under microwave excitation with varying frequency.
