# Supplementary figures and images for: Nonlinear multi-magnon scattering in artificial spin ice
Source: Nat Commun. 2023 Jun 9;14:3419. doi: 10.1038/s41467-023-38992-7 (PMC10256710; doi:10.1038/s41467-023-38992-7)

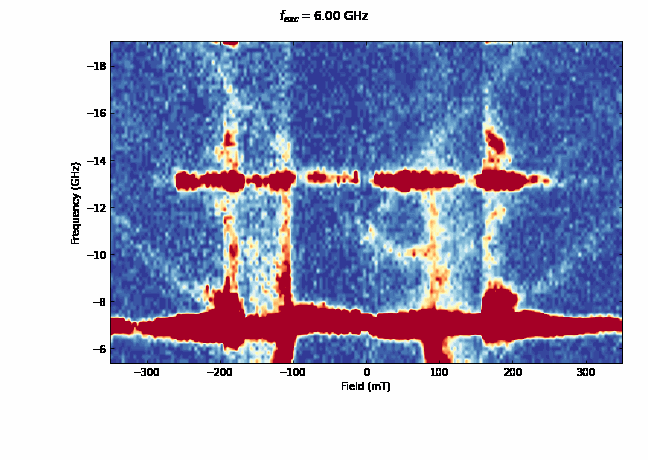

Supplement: Supplementary file 3 — Supplementary Movie 1 [file 41467_2023_38992_MOESM3_ESM.gif]
